# The Quantitative ER Immunohistochemical Analysis in Breast Cancer: Detecting the 3 + 0, 4 + 0, and 5 + 0 Allred Score Cases

**DOI:** 10.3390/medicina55080461

**Published:** 2019-08-10

**Authors:** Ivan R. Ilić, Nikola M. Stojanović, Niko S. Radulović, Vesna V. Živković, Pavle J. Randjelović, Aleksandar S. Petrović, Marina Božić, Ratko S. Ilić

**Affiliations:** 1 Institute of Pathology, Faculty of Medicine, University of Niš, 18000 Niš, Serbia; 2 Faculty of Medicine, University of Niš, 18000 Niš, Serbia; 3 Department of Chemistry, Faculty of Sciences and Mathematics, University of Niš, 18000 Niš, Serbia; 4 Department of Physiology, Faculty of Medicine, University of Niš, 18000 Niš, Serbia; 5 Department of Histology, Faculty of Medicine, University of Niš, 18000 Niš, Serbia; 6 Centre for Radiology, Clinical Center Niš, 18000 Niš, Serbia

**Keywords:** lobular breast cancer, Allred scoring system, estrogen immunopositivity, quantitative analysis

## Abstract

*Background and objectives:* The currently used immunohistochemical approach in determining the estrogen receptor (ER) positivity of breast cancers (BCs) is inherently subjective and additionally limited by its semi-quantitative nature. The application of software in the analysis of digitized slide images may overcome some of these limitations. However, the utilization of such an approach requires that the entire staining procedure is standardized. Background and objectives: We aimed to establish a procedure for the photometric and morphometric analysis of BC immunohistochemical parameters that can possibly be used for a diagnostic purpose that is in line with the current semi-quantitative scoring system. *Materials and Methods:* Semi-quantitative analysis of ER-stained tissue sections was performed following the Allred scoring system guidelines. The quantitative analysis was performed in ImageJ software after color deconvolution. The quantitative analysis of 66 cases of invasive lobular BC included: Percent of ER-positive cells, average nuclear coloration intensity, and the quantitative ER score. The percent of ER-positive tumor cells was counted using a standard grid overlay, while optical density (0.0–1.0) was measured within each nucleus at the grid points. *Results:* A statistical analysis revealed a significant positive correlation (*r* = 0.886, *p* < 0.001) between the subjective semi-quantitative and quantitative ER scores, with a large effect size (*d* = 3.8215). We observed strong statistically significant correlations between individual parameters of the total ER score, percentage of ER-positive nuclei, and color intensity, obtained by the two independent methods. *Conclusions:* Additionally, besides excluding subjectivity, the up to now unreported cases of 3 + 0, 4 + 0, and 5 + 0 Allred scores were detected only by the application of the proposed quantitative approach.

## 1. Introduction

Around 70% of human breast cancers (BCs) express estrogen receptors (ERs) and, based on many demands (diagnosis, therapy, etc.), BCs are divided into estrogen-dependent and independent ones [1]. Estrogen, as a transcription factor, regulates the genetically-programmed progression of cell cycle and growth in mammary glands. Its quantitative expression, which could reflect on both clinical (disease outcome) and laboratory work, has been extensively studied [2,3,4,5,6]. One of the most important prognostic and predictive features of BCs is their ER-positivity, and based on this information the therapeutic choice (hormone therapy), time of diagnosis, and patient survival can be significantly influenced [7,8].

Up to 20% of the results of immunohistochemical analysis of ER around the world can either be false positive (+) or false negative (−) due to variations in pre-analytical variables, the positivity of cutoff values, and criteria of interpretation [9]. Several common tumor characteristics found in a number of false ER (−) BCs include poor fixation, negative (negative ER expression in the present normal ductal epithelium), or absent controls (positivity within the ductal epithelium) [10]. The cases when one should suspect false ER (−) BCs might include tubular, lobular, and mucous histological types with a Nottingham score of 1, i.e., although the current tumor is ER (−), the tumors with the same nuclear grade or Nottingham score are most often ER (+). Today, the ER status is usually obtained by summing the score of the percentage abundance and the staining intensity of ER-stained nuclei of tumor cells (the so-called Allred score, ranging from 0 to 8). Based on the correlation analysis of “cut-point” immunohistochemical scores and patient survival, it was concluded that patients with Allred scores ≥3 (representing 10% of the cells with low ER-positivity), receiving adjuvant endocrine therapy, had a statistically significantly better disease prognosis than those with Allred scores <3 [9].

The usage of immunohistochemical staining has evolved from a mere qualitative special staining procedure to the quantitative one serving as a prognostic/predictive marker [11]. The routinely used immunohistochemical approach has limitations due its the semi-quantitative nature, i.e., the lack of full quantitative potential of the immuno-probe visualization on tissue samples. In order to obtain adequate immunohistochemical results, the utilization of computer-assisted approaches are recommended; however, such an approach requires that the entire staining procedure is standardized [12,13,14,15,16].

Having in mind that the ER-positivity score and patient survival correlate positively [17], and the need for an objective ER quantification, we aimed to establish a procedure for the photometric and morphometric analysis of BC immunohistochemical parameters corresponding to BCs with different biological aggressiveness, that can possibly be used for differential diagnostic purposes that are in line with the current semi-quantitative scoring system. The study was conducted on 66 samples of BC, stained using a standard method for the presence of ER, using a procedure that encompassed image processing and nuclear color intensity quantification.

## 2. Materials and Methods

### 2.1. Patients

Out of all cases diagnosed with BC during a two-year period (2009–2011), 66 cases of invasive lobular carcinomas (ILCs) belonging to the two most common variants (classical or pleomorphic) were chosen for this analysis. Tissue samples of ILC were obtained by breast-conserving surgery or mastectomy with axillary dissection in the Clinical Centre Niš and other clinical centers from south-eastern Serbia. The samples were routinely processed, embedded in paraffin, and archived together with their corresponding histopathological diagnosis and clinical documentation in the Centre of Pathology of the Clinical Centre Niš. The study design was approved by the Ethics Committee (No. 12-3627-2/3) on 14 April 2016.

### 2.2. Immunohistochemical Staining

Immunohistochemical staining was performed for the characteristic areas of tumors (1–2 paraffin blocks per case) from microscopically selected samples (regions), based on standard (hematoxylin and eosin) H&E staining. Tissue from the paraffin molds was cut into 4-µm-thick sections and placed on Superfrost glass slides. Antigen retrieval of deparaffinized and rehydrated samples was done in citric acid buffer (pH 6.0) in a microwave oven for 20 min. After cooling to room temperature, the blockage of endogenous peroxidase was performed using 3% (*w*/*w*) hydrogen peroxide. After sample washing (PBS, pH 7.4), primary estrogen (Monoclonal Mouse Anti-Human Estrogen Receptor α (ER); Clone 1D5; Code N1575, Ready-to-use; Dako, Glostrup Denmark) antibody was applied for 40 min at room temperature in a moist chamber. Visualization was achieved by incubation of slides with Dako LSAB2 System-HRP (Code K0673, 15 mL) and diaminobenzidine (DAB), followed by washing and counterstaining with Mayer’s hematoxylin.

### 2.3. Scoring System

**Figure 1:**
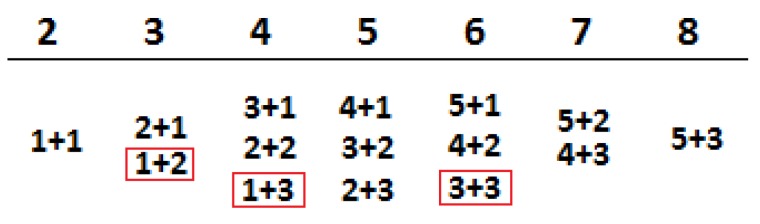
The addend combinations for the Allred scoring system; red circled combinations are those score combinations that were not found in our study or are extremely rare.

Semi-quantitative analysis of ER-stained tissue sections was performed following the Allred scoring system guidelines. To obtain the final scores, individual scores of the percentage of ER-positive cancer cell nuclei (0–5) and the staining intensity of the nuclei (0–3) (Figure 1) were summed up. The percentage of ER-positive cancer cell nuclei was set as follows: 1—less than 1% of positive cancer cell nuclei; 2—from 1 to 10% of positive cancer cell nuclei; 3—from 11 to 33% of positive cancer cell nuclei; 4—from 34 to 66% of positive cancer cell nuclei; and score 5—more than 67% of positive cancer cell nuclei (Figure 1; first addend). Whereas, the staining intensity in the nuclei was scored as: 1—weak; 2—medium; and 3—strong (Figure 1; second addend).

### 2.4. Experimental Scoring System

Tissue sections were observed using BX-50 microscope (Olympus Co., Tokyo, Japan) and images (TIFF) of selected fields captured with a SONY CCD Color Video camera HYPER HAD connected to the microscope. After the adjustment of the Köhler illumination field, the aperture opening (0.3) and illumination were set to a constant value, while all camera options connected with the automatic image corrections (shutter, gain) were switched off and white balance was adjusted to 3200. We used a blue microscope filter and an indifferent filter ND6. The obtained images, saved in TIFF format, without additional corrections were further analyzed using ImageJ software version 1.5 (http://rsb.info.nih.gov/ij/).

**Figure 2:**
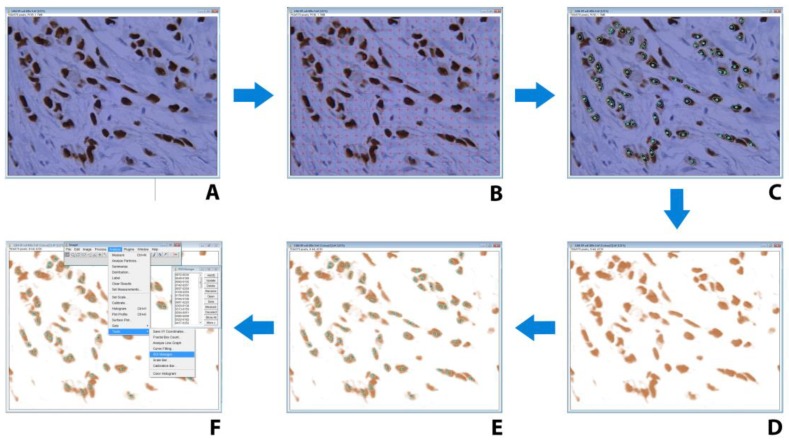
The procedure of region of interest (ROI) selection and color deconvolution in ImageJ software, on digitalized images of microscopic fields of estrogen receptor (ER) immunohistochemistry counterstained with hematoxylin. (**A**) Digitalized microscopic field of ER immunohistochemistry counterstained with hematoxylin. (**B**) Overlaid grid with regularly-distanced crosses (24 × 18) for unbiased selection of nuclei. (**C**) Manual tracing of nuclear contours, which results in saved overlay for further usage as ROI. (**D**) Application of plug-in for image color deconvolution (version 1.5). (**E**) Importing overlay of selected nuclei contours form ROI manager. (**F**) measurement of optical density for nuclei from ROI.

For the analysis of each tissue section, at least 10 fields under 400× magnification were chosen. Fields with normal or dysplastic breast tissue, as well as those with focal lobular breast carcinoma “in situ”, were not analyzed. The precisely-defined grid system was used for the analysis of all images obtained from the studied cases. The outline of each nucleus, found on the grid points, was drawn/made and added/saved in ROI (Region of Interest) manager. Afterward, color deconvolution, based on a Landini algorithm and incorporated as a plugin, was applied to all images and the saved nuclear outlines were overlaid on these modified images (Figure 2). Inside each outlined nucleus, optical density (OD) was measured. For calibration purposes, OD was set to a range from 0 (on a binary image corresponding to almost white, while in our case this was a pale blue hematoxylin coloration) to 255 (almost black). According to the Lambert-Beer law, the OD of each nucleus is directly proportional to the amount of the dye bound for the nuclear structures. Thus, OD = 0 means that there is no dye, OD = 1.0 means that 90% of photons are absorbed, while OD = 2.0 correlates with 99% of absorbed photons [11]. The total number of the measured nuclei per case was taken to correspond to 100%, while the limit between the positive and negative ones was set to 0.1 OD, corresponding to the 10% of DAB OD [18]. The quantitative ER score was assessed as follows:

Quantitative ER score = 1/20 * (percent of ER-positive cancer cell nuclei + (average nuclear intensity × 100)).

### 2.5. Statistical Analysis

The obtained values for the evaluated photometric parameters, as well as the semi-quantitative ER score, were subjected to the following statistical methods: (i) Descriptive statistics (mean (X), standard deviation (SD), median, maximum, and minimum values), (ii) correlation analysis (Pearson test), and (iii) effect size. All statistical analyses were performed using SigmaStat 2.0 (SPSS Inc., Chicago, IL, USA) and GraphPad Prism version 5.03, (San Diego, CA, USA).

## 3. Results

**Figure 3:**
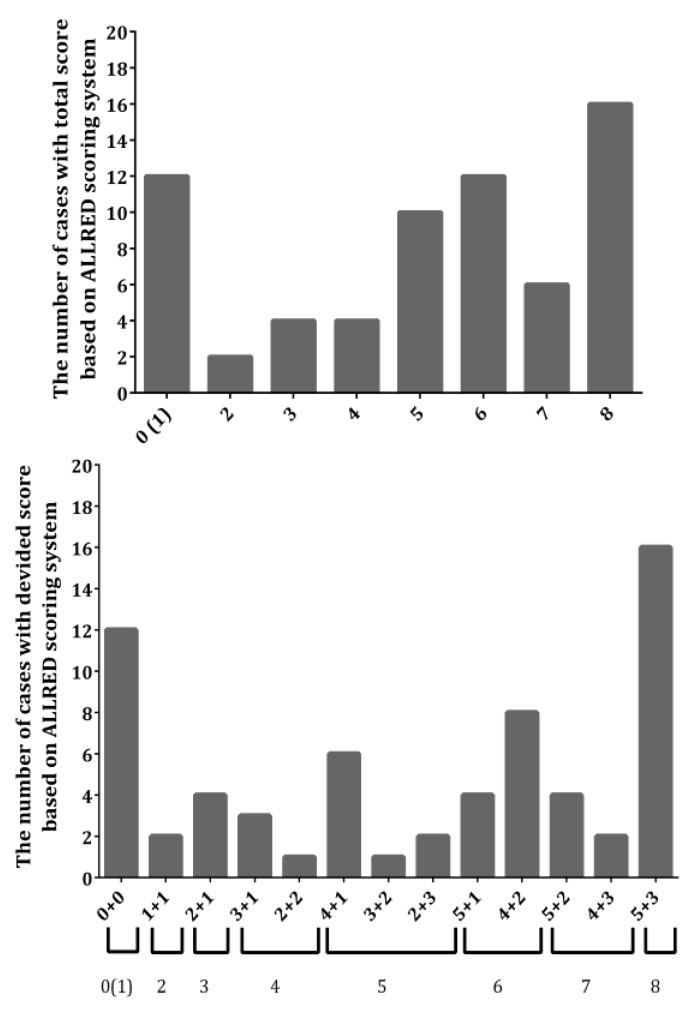
Histograms with the results of the semi-quantitative analyses, providing the distribution of the number of cases utilized in this work based on the total Allred score (**up**) and the breakdown of specific addend combinations of the scores (**down**).

**Figure 4:**
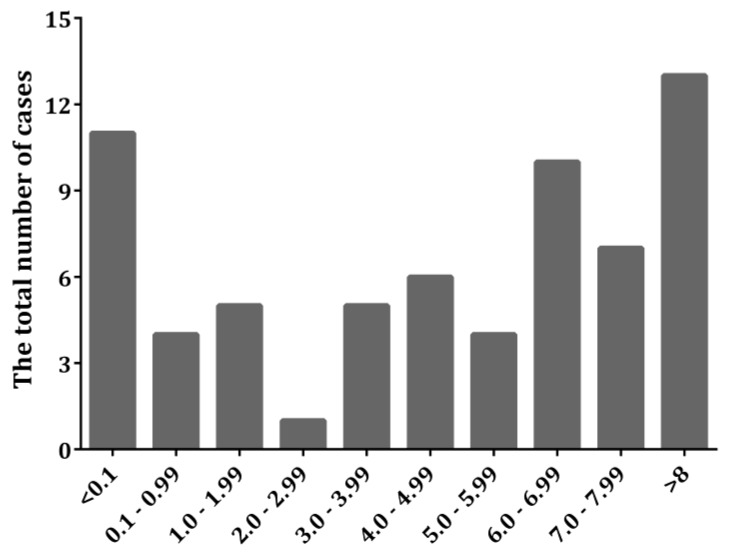
Histogram giving the distribution (number of cases) of the results of the quantitative analyses of the same cases as given in Figure 3.

Both the semi-quantitative and quantitative analyses of the 66 cases of ILC considered in our study included the determination of the following three parameters: Percent of ER-positive cells, average nuclear coloration intensity, and quantitative ER score. In the semi-quantitative, classical approach, the first two parameters were evaluated by at least two experienced pathologists and were based on their subjective treatment of the coloration intensity (which attained integer values, 0–3), while the percentage of the ER-positive nuclei was assessed by manual counting and was scored typified according to the Allred ranges (Figure 3). The proposed quantitative approach in this work included both the counting and coloration intensity evaluation by a software that utilized a deconvoluted color intensity and a counting grid. The results of the quantitative assessment were scaled to be compatible with the already utilized Allred score. Following the subjective semi-quantitative analysis of all of the cases included in the study, we initially considered the ER-negative ones. The deconvoluted color intensity assessment allowed us to detect cases where OD values of 0.1 and less were present in the negative cases (Figure 4). All cases that were determined to have a higher OD of 0.1 were observable by the pathologists. Thus, we chose OD = 0.1 as the limit when the score 0 or 1 for nuclear coloration was given to a case to maintain compatibility with the Allred scoring system. Since the maximum OD value cannot surmount 1 [11], the limit (OD = 0.1) chosen represents the lowest 10% cutoff value.

The histograms in Figure 3 and Figure 4 summarize the descriptive (number of cases and their breakdown into specific score combinations) results of both approaches, and present the frequency of occurrence of a specific value of the parameters within the ranges of the studied cases. As it can be seen from the histograms, the quantitative approach pointed to the existence of ILCs which contained a low percentage of colored nuclei, but the intensity of the coloration was still assignable to the score 1, i.e., to the possibility of the total score 3 + 0, which was hardly, if at all, detected by the naked human eye. There were even cases where the total score was 4 + 0, in cases where a low percentage of colored nuclei were counted but a much greater color intensity was revealed after deconvolution. We observed additional unusual cases where the background blue staining masked the brow coloration of the positive nuclei; hence, these were not accounted for by a simple visual inspection by the pathologists, but were clearly detectable after deconvolution by the software. Surprisingly, these cases would be classified as belonging to the 3 + 0, or even 4 + 0 and 5 + 0, total Allred scores (Table 1).

**Figure 5:**
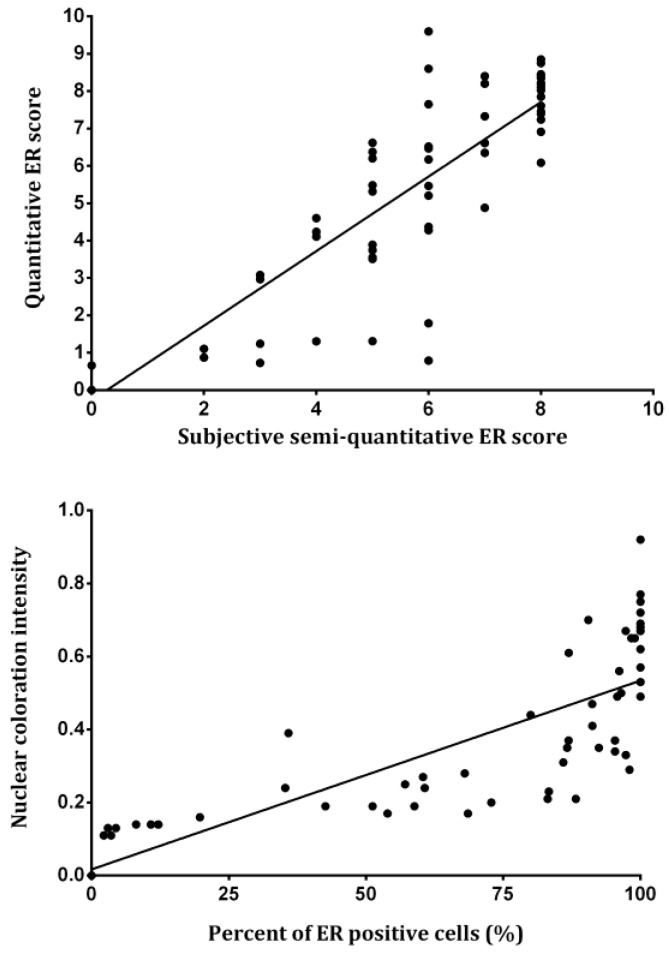
The correlation between the subjective semi-quantitative and the quantitative ER scores (**up**), and the correlation between the quantitative nuclear stain intensity and the percent of ER-positive nuclei (**down**).

For the rest of the cases assessed in this work, there wa excellent correlation between the semi-quantitative and the quantitative approach results. The correlation analysis performed on all cases revealed a statistically significant positive correlation (*r* = 0.886, *p* < 0.001) between the subjective semi-quantitative and the quantitative ER scores, with a large effect size (*d* = 3.8215) (Figure 5, up). Please note the cases with low OD values in the plot (quantitative score), i.e., the ones that do not fit this good correlation of the remaining cases. These cases represent instances of a score overestimation by the pathologists (Figure 5, up), hence the quantitative approach provides a means of detecting human errors.

The correlations between individual parameters of the total ER score, percentage of ER-positive nuclei, and color intensity obtained by two independent methods are presented in Table 2. The results show that both parameters have strong statistically significant correlations.

Inherently, the color intensity of the ER-positive nuclei was in direct positive connection with the number of these nuclei, and the lower laying cases of low-intensity coloration are the problematic ones, as stated above for the subjective semi-quantitative assessment. The correlation analysis revealed a statistically significant positive correlation (*r* = 0.719, *p* < 0.001) between the quantitative nuclear coloration intensity and the percent of ER-positive nuclei (Figure 5, down). However, since the Allred system ranges for the percentage of ER-positive nuclei have an uneven distribution, the scores take higher values for smaller increments at first, and are considered maximum after 67%; if we were to exclude this final score, the correlation would become much higher. The noted correlation (Figure 5, down) between the percentage of ER-positive nuclei and the stain intensity is in agreement with the general rare occurrence of cases of 1 + 2 and 1 + 3 (Figure 1), as these were not detected at all in our sample.

## 4. Discussion

The standardization of immunohistochemical staining procedures, making them more adequate and routine, is still debatable. Besides these laboratory-oriented issues, the question of stained tissue interpretation has become important for BC therapy outcome. Quantitative immunohistochemistry protocols require following an appropriate procedure during tissue sample processing and image analysis [11,19]. The usage of the quantitative immunohistochemistry, based on single pixels in cells/tissue, is quite difficult in everyday clinical practice, due to the variations in tissue sampling, their further processing, and analysis. The best way to avoid these discrepancies is an automation of all mentioned processes [19]. Although immunohistochemical staining has numerous advantages, there are still no definite solutions for the standardization and interpretation of results [20]. The procedure itself can be easily standardized; however, the interpretation of the results is based only on a visual subjective scoring system [21]. A great number of pathologists differentiate positive and negative immunohistochemical results according to a subjective qualification of positivity and percentage abundances, where the defined limits are between 5 and 45% [22].

Immunohistochemical semi-quantitative assessment of ER expression, for the purposes of therapy inclusion, is recommended by the American Society of Clinical Oncology (ASCO) [23]. Semi-quantitative procedures have both inter- and intra-observer variations [24]; however, some studies have shown that they are still useful for the evaluation of biopsy samples and have significance in an everyday clinical practice [25,26,27]. Their major downfall is in the evaluation of border-line cases of positivity; thus, it is of vast importance to improve them in terms of steroid receptor positivity analysis [28]. There are publications that emphasize that 80% of laboratories show positive ER with medium/strong expression, while only in 37% with weak expression of ER positivity [29]. Several publications applied semi-quantitative scores for the estimation of nuclear staining as a direct connection to the number of ER in cells [27]. However, these systems are with high levels of subjectivity, and inter-observer variations are still present [21]. In order to ensure data standardization, different software was used and a significant correlation with semi-quantitative scores and biochemical parameters was found [30,31,32,33]. Nevertheless, the complexity and high pricing are major limitations for the application of software in routine diagnostics.

Adequately stained tissue sections with monoclonal antibodies have a two-colored character, where the brown coloration arises from the antibody-bound structures (nucleus in our case) while the blue is due to a non-specific hematoxylin background staining affecting all tissue structures. These premises were taken into consideration in our experimental design, where the image color deconvolution was applied (Figure 5). Previous methods have tried to overcome this color-related issue by subtraction of color intensity of the nucleus with that of the background [21]. The problem with this technique is that the difference between the dark blue and light brown nuclei cannot be made; this is avoided in our study by separating signals of DAB-staining from those originating from hematoxylin (deconvolution). This deconvolution algorithm has been previously suggested by Ruifrok and Johnston for the same purposes as presented here [34].

We focused our study on the analysis of immunohistochemically-stained characteristic areas of tumors (periphery of the sample), based on standard H&E staining. The analysis of these areas seems to be the most adequate one, since a number of researchers have found different ER expression in the central parts compared to the periphery of the tumorous mass [35]. This is of great importance since, in some cases, the heterogeneous expression of ER can be observed [36]. Also, the analyzed ER expression seemed better-compared to the progesterone receptor expression due to the relatively equal expression of progesterone receptors in the surrounding cells [7]. Besides the ER expression, these areas are characterized by different morphological features of tumor cells and have a larger value of the proliferative index than the central parts [28]. Although we cannot eliminate the subjective nuclei labeling during the analysis, insertion of a gird with a predetermined number of grid points partially decreases this form of subjectivity. Strong positive correlation between the subjective semi-quantitative and quantitative ER scores (Figure 5, down, *r* = 0.886) suggests that the approach in which 10 fields at the periphery parts of tumor mass are chosen during the analysis might be adequate for the estimation of ER expression.

One of the major features of this study is that the analysis is based on simple, cheap, and available software: ImageJ. Finally, it represents a great benefit for patients with BC, since receptor expression analysis is crucial for the determination of the prognostic indexes [25]. Although no generally accepted standards for morphometric and photometric analysis are available [37], one can say that the results of this study can be useful for comparison between different histological variants of BC. The Allred scoring system is based on the semi-quantitative estimation of percentage abundance of positive cancer cell nuclei and staining intensity, where the expression limit is 10% of weakly or 1% of medium-stained cancer cell nuclei [18]. However, different previous studies have estimated a cutoff value between positive and negative immunohistochemical staining [38,39]. A shortcoming of the Allred scoring system exists, since some of the possibilities for the final score are only hypothetical (3 + 0, 4 + 0, and 5 + 0) (Table 1). One may say that our study overcomes such shortcomings based on the strong positive correlation between the quantitative nuclear stain intensity and the percent of ER-positive nuclei (Figure 5, down).

## 5. Conclusions

There are two major beneficial points in the currently proposed approach; the first one giving the possibility to detect cases of stain intensity that were obscured by the background coloration, and the second, to eliminate the overestimation of the score values by pathologists. The comparison and evaluation of semi-quantitative scoring systems (such as the Allred scoring system) are necessary for the standardization of quantitative ER expression assessment. Thus, the suggested deconvolution method can distinguish different variants of ILC, reduce intra-laboratory variations, and exclude subjectivity during ER analysis. Also, the application of the deconvolution method can be useful for the detection of border-line ER (+) cases which reflects directly on hormonal therapy usage.

## Figures and Tables

**Table 1:** The specific stain intensities and percentages of ER-positive nuclei for the cases where the Allred score 0 was allocated based on the semi-quantitative analysis.

Number of Cases	Stain Intensity	Percent of ER-Positive Nuclei (%)
Case No1	0.00015836	55
Case No2	0.00866000	82
Case No3	0.00002071	14
Case No4	0.00340193	78
Case No5	0.00838533	73
Case No6	0.00037859	73
Case No7	0.00006853	27
Case No8	0	0
Case No9	0.0001780	65
Case No10	0.0055402	98
Case No11	0.0000863	57
Case No12	0.0018610	99
Total (average ± SD)	0.00240 ± 0.00334	60 ± 31

**Table 2:** Correlation between parameters obtained using our experimental scoring system and Allred scoring system.

Parameter	*R* ^2^	Correlation Magnitude	*p*-Value
% of ER-positive cancer cell nuclei	0.741	Strong	<0.001
Stain intensity in the nuclei	0.638	Strong	<0.001

## References

[1] Lacroix M., Leclercq G. (2004). About GATA3, HNF #A and XBP, three genes co-expressed with estrogen alpha gene (ESR1) in breast cancer. Mol. Cell. Endocrinol..

[2] Dieci M.V., Piacentini F., Dominici M., Omarini C., Goubar A., Ficarra G., Conte P., Guarneri V. (2014). Quantitative expression of estrogen receptor on relapse biopsy for ER-positive breast cancer: Prognostic impact. Anticancer Res..

[3] Campbell F., Elston C., Blamey R., Morris A., Nicholson R., Griffiths K., Haybittle J. (1981). Quantitative oestradiol receptor values in primary breast cancer and response of metastases to endocrine therapy. Lancet.

[4] McClelland R.A., Berger U., Miller L.S., Powles T.J., Jensen E.V., Coombes R.C. (1986). Immunohistochemical assay for estrogen receptor: Relationship to outcome of therapy in patients with advanced breast cancer. Cancer Res..

[5] Ma H., Lu Y., Marchbanks P.A., Folger S.G., Strom B.L., McDonald J.A., Simon M.S., Weiss L.K., Malone K.E., Burkman R.T. (2013). Quantitative measures of estrogen receptor expression in relation to breast cancer-specific mortality risk among white women and black women. Breast Cancer Res..

[6] Kamby C., Rasmussen B.B., Kristensen B. (1989). Oestrogen receptor status of primary breast carcinomas and their metastases. Relation to pattern and spread and survival after recurrence. Br. J. Cancer.

[7] Szeszel M.K., Crisman C.L., Crow L., McMullen S., Major J.M., Natarajan L., Saquib A., Feramisco J.R., Wasserman L.M. (2005). Quantifying Estrogen and Progesterone Receptor Expression in Breast Cancer by Digital Imaging. J. Histochem. Cytochem..

[8] Fisher B., Redmond C., Fisher E.R., Caplan R. (1988). Relative worth of estrogen or progesterone receptor and pathologic characteristics of differentiation as indicators of prognosis in node negative breast cancer patients: Findings from National Surgical Adjuvant Breast and Bowel Project Protocol B-06. J. Clin. Oncol..

[9] Hammond M.E.H., Hayes D.F., Wolff A.C., Mangu P.B., Temin S. (2010). American Society of Clinical Oncology/College of American Pathologists Guideline Recommendations for Immunohistochemical Testing of Estrogen and Progesterone Receptors in Breast Cancer. J. Oncol. Pract..

[10] Cameron M.A. (2009). Commision of Inquiry on Hormone Receptor Testing.

[11] Taylor C.R., Levenson R.M. (2006). Quantification of immunohistochemistry—Issues concerning methods, utility and semiquantitative assessment II. Histopathology.

[12] Grube D. (2004). Constants and variables in immunohistochemistry. Arch. Histol. Cytol..

[13] Diaz L.K., Sneige N. (2005). Estrogen receptor analysis for breast cancer: Current issues and keys to increasing testing accuracy. Adv. Anat. Pathol..

[14] Battifora H., Seidal T., Balaton A.J. (2001). Interpretation and Quantification of Immunostains. Am. J. Surg. Pathol..

[15] Umemura S., Itoh J., Itoh H., Serizawa A., Saito Y., Suzuki Y., Tokuda Y., Tajima T., Osamura R.Y. (2004). Immunohistochemical evaluation of hormone receptors in breast cancer: Which scoring system is suitable for highly sensitive procedures?. Appl. Immunohistochem. Mol. Morphol..

[16] Umemura S., Osamura R.Y. (2004). Utility of immunohistochemistry in breast cancer practice. Breast Cancer.

[17] Bae S.Y., Kim S., Lee J.H., Lee H.-C., Lee S.K., Kil W.H., Kim S.W., Lee J.E., Nam S.J. (2015). Poor prognosis of single hormone receptor- positive breast cancer: Similar outcome as triple-negative breast cancer. BMC Cancer.

[18] Badve S.S., Baehner F.L., Gray R.P., Childs B.H., Maddala T., Liu M.-L., Rowley S.C., Shak S., Perez E.D., Shulman L.J. (2008). Estrogen- and Progesterone-Receptor Status in ECOG 2197: Comparison of Immunohistochemistry by Local and Central Laboratories and Quantitative Reverse Transcription Polymerase Chain Reaction by Central Laboratory. J. Clin. Oncol..

[19] Taylor C.R. (2006). Quantifiable internal reference standards for immunohistochemistry: The measurement of quantity by weight. Appl. Immunohistochem. Mol. Morphol..

[20] Lin F., Chen Z. (2014). Standardization of Diagnostic Immunohistochemistry: Literature Review and Geisinger Experience. Arch. Pathol. Lab. Med..

[21] Lehr H.-A., Mankoff D.A., Corwin D., Santeusanio G., Gown A.M. (1997). Application of Photoshop-based Image Analysis to Quantification of Hormone Receptor Expression in Breast Cancer. J. Histochem. Cytochem..

[22] Battifora H., Mehta P., Ahn C., Esteban J. (1993). Estrogen receptor immunohistochemical assay in paraffin-embedded tissue. A better gold standard?. Appl. Immunohistochem..

[23] Bast R.C., Ravdin P., Hayes D.F., Bates S., Fritsche H., Jessup J.M., Kemeny N., Locker G.Y., Mennel R.G., American Society of Clinical Oncology Tumor Markers Expert Panel (2001). Update of recommendations for the use of tumor markers in breast and colorectal cancer. Clinical practice guidelines of the American Society of Clinical Oncology. J. Clin. Oncol..

[24] Jagoe R., Steel J.H., Vucicevic V., Alexander N., Van Noorden S., Wootton R., Polak J.M. (1991). Observer variation in quantification of immunocytochemistry by image analysis. J. Mol. Histol..

[25] Schnorrenberg F., Pattichis C., Kyriacou K., Schizas C., Pattichis C. (1997). Computer-aided detection of breast cancer nuclei. IEEE Trans. Inf. Technol. Biomed..

[26] Zhong F., Bi R., Yu B., Yang F., Shui R. (2016). A Comparison of Visual Assessment and Automated Digital Image Analysis of Ki67 Labeling Index in Breast Cancer. PLoS ONE.

[27] Turbin D.A., Leung S., Cheang M.C., Kennecke H.A., Montgomery K.D., McKinney S., Treaba D.O., Boyd N., Goldstein L.C., Badve S. (2008). Automated quantitative analysis of estrogen receptor expression in breast carcinoma does not differ from expert pathologist scoring: A tissue microarray study of 3484 cases. Breast Cancer Res. Treat..

[28] Wied G.L., Bartels P.H., Bibbo M., Dytch H.E. (1989). Image analysis in quantitative cytopathology and histopathology. Hum. Pathol..

[29] Rhodes A., Jasani B., Barnes D., Bobrow L., Miller K. (2000). Reliability of immunohistochemical demonstration of oestrogen receptors in routine practice: Interlaboratory variance in the sensitivity of detection and evaluation of scoring systems. J. Clin. Pathol..

[30] Sklarew R.J., Bodmer S.C., Pertschuk L.P. (1990). Quantitative imaging of immunocytochemical (pap) estrogen receptor staining patterns in breast cancer sections. Cytometry.

[31] Baddoura F.K., Cohen C., Unger E.R., DeRose P.B., Chenggis M. (1991). Image analysis for quantification of estrogen receptor in formalin-fixed paraffin-embedded sections of breast carcinoma. Mod. Pathol..

[32] Aziz D.C. (1992). Quantitation of estrogen and progesterone receptors by immunocytochemical and image analysis. Am. J. Clin. Pathol..

[33] Auger M., Katz R.L., Johnston D.A., Sneige N., Ordonez N.G., Fritsche H. (1993). Quantitation of immunocytochemical estrogen and progesterone receptor content in fine needle aspirates of breast carcinoma using the SAMBA 4000 image analysis system. Anal. Quant. Cytol. Histol..

[34] Ruifrok A.C., Johnston D.A. (2001). Quantification of histochemical staining for color deconvolution. Anal. Quant. Cytol. Histol..

[35] Douglas-Jones A.G., Collett N., Morgan J.M., Jasani B. (2001). Comparison of core oestrogen receptor (ER) assay with excised tumour: Intratumoral distribution of ER in breast carcinoma. J. Clin. Pathol..

[36] Saria E., Mooney E.E., Liu K., Dodge R.R., Layfield L.J. (1998). Tissue Heterogeneity of Immunohistochemically Detected Estrogen Receptor: Implications for Image Analysis Quantification. Am. J. Clin. Pathol..

[37] Ikpatt O.F., Kuopio T., Collan Y. (2002). Nuclear morphometry in African breast cancer. Image Anal. Stereol..

[38] Dowsett M., Bartlett J., Ellis I.O., Salter J., Hills M., Mallon E., Watters A.D., Cooke T., Paish C., Wencyk P.M. (2003). Correlation between immunohistochemistry (HercepTest) and fluorescence in situ hybridisation (FISH) for HER-2 in 426 breast carcinomas from 37 centres. J. Pathol..

[39] Allred D.C., Clark G.M., Elledge R., Fuqua S.A., Brown R.W., Chamness G.C., Osborne C.K., McGuire W.L. (1993). Association of p53 protein expression with tumour cell proliferation rate and clinical outcome in node-negative breast cancer. J. Natl. Cancer Inst..

